# Let the Children Listen: A First Approximation to the Sound Environment Assessment of Children through a Soundwalk Approach

**DOI:** 10.3390/ijerph17124185

**Published:** 2020-06-12

**Authors:** Laura Estévez-Mauriz, Jens Forssén, Georgios Zachos, Wolfgang Kropp

**Affiliations:** Division of Applied Acoustics, Department of Architecture and Civil Engineering, Chalmers University of Technology, SE-412 96 Gothenburg, Sweden; jens.forssen@chalmers.se (J.F.); georgios.zachos@chalmers.se (G.Z.); wolfgang.kropp@chalmers.se (W.K.)

**Keywords:** children, urban sound planning, environmental noise, soundwalk, built environment, field studies

## Abstract

The urban sound environment is one of the layers that characterizes a city, and several methodologies are used for its assessment, including the soundwalk approach. However, this approach has been tested mainly with adults. In the work presented here, the aim is to investigate a soundwalk methodology for children, analyzing the sound environment of five different sites of Gothenburg, Sweden, from children’s view-point, giving them the opportunity to take action as an active part of society. Both individual assessment of the sound environment and acoustic data were collected. The findings suggested that among significant results, children tended to rank the sound environment as slightly better when lower levels of background noise were present ($L_{A90}$). Moreover, traffic dominance ratings appeared as the best predictor among the studied sound sources: when traffic dominated as a sound source, the children rated the sound environment as less good. Additionally, traffic volume appeared as a plausible predictor for sound environment quality judgments, since the higher the traffic volume, the lower the quality of the sound environment. The incorporation of children into urban sound environment research may be able to generate new results in terms of children’s understanding of their sound environment. Moreover, sound environment policies can be developed from and for children.

## 1. Introduction

Urban space users are shaping the environment; therefore, they are active users. In this framework, listening is not a passive sense, and the study of urban sound environment requires integration within the holistic analysis of the urban planning process, leading to an “urban sound planning” approach [1] as part of the multi-sensorial approach to the urban experience. In the urban space configuration, where space influences human experience and behavior [2], the analysis goes beyond noise control; field studies involving active listening, i.e., that the listener is looking for empathy and understanding [3], concentrated on interpreting what is happening in the surroundings using a series of acquired tools, become an essential part to evaluate the sound environment and understand how it is perceived. In this type of active listening, children are normally excluded; however, they are often considered as a group at risk [4] in terms of noise exposure.

### 1.1. Environmental Inequality of Noise on Children

The negative effects of a certain noise exposure may be larger for one group of people than for another group, which may be called the group’s vulnerability level [5]. The vulnerability level for children as a group is higher since they may have more difficulties recognizing dangerous noise exposures [6], which means less ability to understand noise stressors that interfere with communication and affect cognitive impairment, coupled with a lack of ability to control their surrounding environment [7,8] at a crucial stage in their development [9].

Children’s vulnerability can also be influenced by noise exposure variation due to social inequalities [10]. Children with a low socio-economic position may be exposed to an accumulation of multiple environmental risks [11], including noise.

A systematic review by Clark and Paunovic on environmental noise and its effect on children’s cognition argued that robust studies are needed [12]. In this sense, among European children, environmental inequalities have been proven; however, the large range of methodologies used in the numerous studies makes it difficult to draw solid conclusions [10].

### 1.2. Hints on Noise Policies and Children

At the Fourth Ministerial Conference on Environment and Health held in 2004, a large number of World Health Organisation (WHO) Member States recognized the need to take measures regarding environmental quality, specifically among children [13]. This Conference laid the foundations for the report on “Protecting the health of children in a changing environment”. One of its objectives was dedicated to the prevention of diseases arising from physical environments, looking for actions to reduce exposure to noise of children, e.g., traffic in residential areas. For this, the authors of the report requested and offered assistance to develop adequate noise guidelines [14]. However, much remains to be done. The recently published report “A future for the World’s Children?”, signed by a Commission of 40 experts in child and adolescent health, states that “no single country is adequately protecting children’s health, their environment and their futures”, urging to place children at the center of efforts related to sustainable development, as well as incorporating their voices in policy decisions, among others [15].

With regard to noise exposure, the risks have been largely studied with respect to wellbeing and cognitive impairment, urging the recognition of the susceptibility of children over adults to certain environmental risk factors and the incorporation of policy recommendations [8,16]. Nevertheless, most of the legislation is still based on data from and for adults.

The WHO has estimated noise damage to health through the index DALYs, which measures the Disability-Adjusted Life-Years; in the children’s case, 45,000 DALYs are lost due to cognitive impairment, meaning 45,000 lost years of healthy life among European children every year [17]. Although there are no additional consequences of these numbers in policies, the Environmental Noise Directive [18] includes an annex allowing the presentation of specific dose-effect relationships in “vulnerable groups of population”, such as children. However, no specific reference to dose-effect relationships in children was found in any of the documents submitted to the European Environment Agency on noise in 2018, known as “EEA noise country fact sheets” [19]. It can be argued that a better understanding by the public of sound exposure is needed [20].

Current environmental health protection is based on a “command-control environmental policy” that refers to objective criteria [21]. However, an explicit inclusion of children is desired. This would guarantee a minimum quality and wellbeing regarding the sound environment for children. However, going further toward the incorporation of subjective criteria capable of addressing contextual factors and increasing awareness is also pursued [22]. Moreover, the interference between the subjective perception and the objective evaluation used by legislation has been studied [22], leading to a constant search for tools and approaches that can facilitate such evaluations. The approach presented here seeks to make children become active listeners of their surrounding environments.

### 1.3. Studies on Children’s Experience in the Sound Environment

In addition to the intrinsic complexity of research on children participating in sound studies, additional ethical aspects may make such studies scarce. In any case, most sound studies in children mainly look at the effect on their health and psychological impairment. One of the main inclusions of children in the sound environment studies was made through the study of noise exposure at school [23,24], assessing mainly the noise impact through the annoyance response of children [25] and the detriment of cognitive skills such as reading [26], speech perception, and listening comprehension [27,28]. Other cognitive impairment outcomes include behavioral changes linked with motivation, memory, language mastery, attention, and frustration [29,30,31]. In addition, the impact of road traffic noise on children’s blood pressure has been highlighted [32]. Throughout this research, agreement on the need for an integrative approach to study the effect of noise on children is clear [33].

Looking from another side to the relationship between children and the sound environment, there are other types of studies that try to involve children in active listening, although they do not extend among the scientific community. An interesting work in this line was the one carried out by the artistic collective “Trames”, named “PAYSages Sonores” (more information can be found at www.paysages-sonores.ca). They are looking toward a creative process of children from different countries that facilitates the exploration of and reflection on sounds and the children’s capacity to appropriate the environment. The intention is to support children to be an active part in society by using sound tools to navigate in space and time.

### 1.4. Soundwalk as a Tool in Assessing the Qualities of the Sound Environment

There is no general agreement on how to collect data and assess the qualities of the sound environment. Efforts to measure and evaluate the sound environment systematically have recently been investigated through the ISO working group on the study of soundscape [34,35]. However, during the last few years, several protocols have been developed and used with the intention to study the sound environment experience [36,37,38,39,40], making use of laboratory and/or in situ experiments, gathering information through surveys, including, e.g., perceived sound source identification, classification of soundscape quality, sound-level measurements, and binaural recordings.

Soundwalks have been widely used as a tool in assessing the perceived urban sound environment. They have also been used by several researchers and practitioners for developing methodologies, e.g., for engaging professionals in urban planning [38] and for characterizing the perceived soundscape quality of residential urban areas, with the intention to move away from the negative value judgment in the study of the sound environment (e.g., unwanted sounds), helping in the development of a methodology to assess exciting and/or restorative soundscapes [40]. Hence, the soundwalk is a concept that has been used in various ways depending on the interests, e.g., addressing how the soundwalk is to be performed, who is performing it, and for what purpose. What interests us the most is the possibility the soundwalk offers to improve current and future planning of urban sites [1,39,41,42,43]. The essence of the soundwalk is to evaluate the sound environment in situ.

Originally, soundwalks had an educational purpose: they intended to encourage the practice of listening [44,45]. They were originally carried out mainly in rural areas [45], whereas today, soundwalks mostly take place in urban areas [36,38,39,40,46,47,48,49]. The concept has been adapted to better understand people’s perception of the urban sound environment [38]. Additionally, Steele et al. summarized a series of positive aspects that a soundwalk has from the participants’ point of view: the learning benefits of an immersive activity, the ability to raise awareness of diverse sound environments, active listening, and the demonstration of the reciprocal influences between the sound environment and the urban design [49].

A large number of researchers use group soundwalks with the intention to collect data from a particular group of people. Typically, a route is designed along a set of locations previously chosen by the organizing team. Examples of this can be found in the soundwalks that are systematically carried out by the group “Sounds in the city” from Montreal, Canada (www.sounds-in-the-city.org), or the “urbanidentity” group in Zurich, Switzerland, which uses soundwalks and workshops to start discussions on urban (acoustic) qualities (more information can be found at www.urbanidentity.info). Furthermore, a vast number of scientific materials has been using soundwalks also for establishing methodologies, e.g., [38,39,46,47,49]. During the evaluation, participants are invited to listen actively to the environment, rating the sites mainly through questionnaires that gather respondents’ impressions. Researchers typically perform audio recordings and/or sound level measurements at the selected locations. The intention is that the measurement data and the questionnaires support each other, establishing a correlation between physical measurement data and people’s assessment of the sound environment [39,40,43,50]. Here, the main trend has been to focus on the link between the perceived annoyance and the overall noise level. Others have attempted to study the interaction between acoustic measurement data and descriptors, apart from noise annoyance, such as the “pleasantness”, “quietness” or “appropriateness” of the sound environment; however, no clear conclusion has been found [51]. Other researchers, such as Schulte-Fortkamp [52], have incorporated psychoacoustic indicators to go further in understanding participant’s listening experience. The need to investigate indicators of sound source audibility has also been suggested [53].

Considering all this, it is clear that there is no agreement on a fixed protocol to perform and evaluate a soundwalk [54]. The protocols vary in many aspects, for example: questions (the type of questions, scale used, normally numerical and/or verbal, the number of questions, etc.), the nature of the participants (selected group, users of the area, etc), the time participants spend at each location and total duration, the type of audio recordings and sound level measurements, as well as their duration, other data collected (visual, sensorial, etc), instructions given to the participants, and the number of locations.

There are several concepts that are usually included in the soundwalk field surveys, most of the time posed as close-ended questions: (1) frequency of site visit and other demographic data; (2) identification of perceived sounds’ sources and their dominance; (3) acoustic comfort: evaluation of the quality and appropriateness of the sound environment; (4) loudness and unpleasantness; and (5) perception descriptors (based on [55]), including “pleasant”, “chaotic”, “vibrant”, “uneventful”, “calm”, “annoying”, “eventful”, and “monotonous” [36,38,56,57]. Other researchers have included as well overall and visual quality aspects [58]. In addition, semi-structured interviews have been conducted with the intention of obtaining detailed explanations trying to understand how participants feel about a particular location or what is most to their liking [38]. There have also been researchers that have gone further to open-ended questions based on verbal description analysis that could identify sound quality criteria and environmental sound categories [59]. These types of interviews and open-ended questions are conducted rather in studies of the urban environment and usually not during soundwalks.

Apart from looking at quality, the appropriateness of the sound environment to a place has been studied, in the sense of fitness to that place. Most of the time, appropriateness has been related to sound environment quality [36,60]. However, there are arguments against this, claiming that a poor sound environment may be appropriate (e.g., a very busy street area might be appropriately noisy) [37], and further study is probably needed. From another point of view, appropriateness has also been discussed concerning its relation to potential or current activities [61]. In this sense, the role of activity as a mediator of the sound environment assessment by users has recently been explored [48,62].

#### Children’s Communication of Reactions to Sound

Soundwalks provide information on communicating emotional states as reactions to sound in a contextualized outdoor environment. In this regard, our emotions are considered essential in the environment perception [63]. The communication of emotional states has been widely measured by affective reactions to stimuli, such as sounds [64,65,66].

As mentioned above, numerical and/or verbal scales are most commonly used in soundwalk studies [54]. Other fields within acoustics that explore emotional reactions to auditory stimuli (e.g., [64,67]) and more recently studying the perception of the sound environment [68] have been using non-verbal methods. One of the most widely used non-verbal scales is the one developed by Bradley and Lang to study emotional responses to an event [69,70]. They considered three factors central to emotional response. These factors, called dimensions, were named as: valence, understood as perceived pleasantness (positive/negative); arousal, representing the level of excitement arising from stimuli (high/low); and dominance, as the ability of the perceived stimuli to capture attention and its assertiveness (total/null). Therefrom, they developed the Self-Assessment Manikin (SAM) as a picture-oriented scale for a questionnaire measuring emotional responses. Three pictorial scales were obtained, one for each dimension, each with five graphic figures, representing emotions communicated by facial expressions or body reactions. Assessing emotions with children implicitly involves the use of non-verbal pictorial scales. SAM is presented as a useful instrument to assess the subjective experience of emotion connected to stimuli in children [70]. There is growing evidence from research using SAM, reflecting a consistent pattern in children’s responses [71,72,73,74]. Other pictorial techniques have been used in studies regarding noise reactions of children with similarly ranging faces: friendly to irritated [75].

## 2. Objectives

In the present study, the interest lies in studying the regular ambient sound environment of the neighborhood to which children are normally exposed. We strongly argue that, when it comes to our common urban surroundings and environmental assessment, children shall be considered and included as an active part of society. As stated by the United Nations Children’s Fund, UNICEF, in the report about the need for an adequate environment for the fulfillment of children’s rights [76], no enjoyment of health is possible within a contaminated environment.

Children’s environmental awareness is based on “learning to see and learning to take action” through formal and informal participation in their community life [77] (p. 99). When children are more aware about their surroundings through training and education, they feel co-responsible in the assessment and development of their cities. One of the main conditions to support such environmental awareness, highlighted by ecological psychology [77], is the “perceptual learning to notice and value the environment”. Another is to provide “opportunities to take responsible roles in community settings” [77] (p. 102), aligning with the principle of co-responsibility that leads to close cooperation within their own community formed by citizens, stakeholders, and public authorities [78]. This will not just leave a good impression on the children, but can be the seed for responsible citizenship, involved in societal debates and committed with their surroundings. Understanding their needs and letting them be part of the evolution and expansion of our communities are crucial in the urban development [79].

The goal of the present pilot study is to test a soundwalk methodology that analyzes the sound environment of different sites from the children’s point of view. Thereby, the children have the opportunity to contribute to present and future urban development, focusing on urban sound planning. Furthermore, the intention is to make them feel part of a project, where they are the main actors. The main questions revolve around these concepts, as follows:

- For the present study, are acoustic indicators capable of describing children’s perception of the sound environment?
- Does the type of sound source influence the evaluation of the sound environment? If so, what type of sound source?
- Based on the results of the soundwalk, can we use the questions that we normally use in the adult questionnaires?

## 3. Materials and Methods

### 3.1. Study Framework and Children Group

As part of a European project, the authors of this paper worked closely with the Municipality of Gothenburg. Part of the work, developed in collaboration with the Environmental Office, was directed toward the accomplishment of a series of activities related to the sound environment as part of the event called “Framtisdveckan” (Future week). This event is well known and takes place every year in different cities in Sweden. The idea is to make visible the transformation toward an ecologically, economically, and socially sustainable city. During this event, a series of actions take place in the city. Our contribution was to give the opportunity for the citizens to listen to their surroundings and rediscover their sense of hearing. This was made through a series of soundwalks in different districts and at different times of the day.

As part of these events, the idea of incorporating a group of children emerged from the meeting with the representative of the Majorna-Linné district of Gothenburg. In this neighborhood, a group of children and their teacher have been participating in workshops on how to improve the development of new urban areas in their neighborhood. During these workshops, the children studied different areas of their neighborhood. Later on, they made models and provided ideas and suggestions on future development plans. The workshop was framed in the dialogue process of the “Älvstaden” (River city) vision of the city [80].

The group of participants consisted of 30 children between 11 and 12 years old attending fifth grade at a school in Gothenburg. The school is located in a central area of the city belonging to the Majorna-Linné district. To make the children experience the whole process, feedback was made through a class presentation to the group of children on general concepts about sound, and in particular about the soundwalk they performed and the results obtained.

### 3.2. Survey

**Figure 1:**
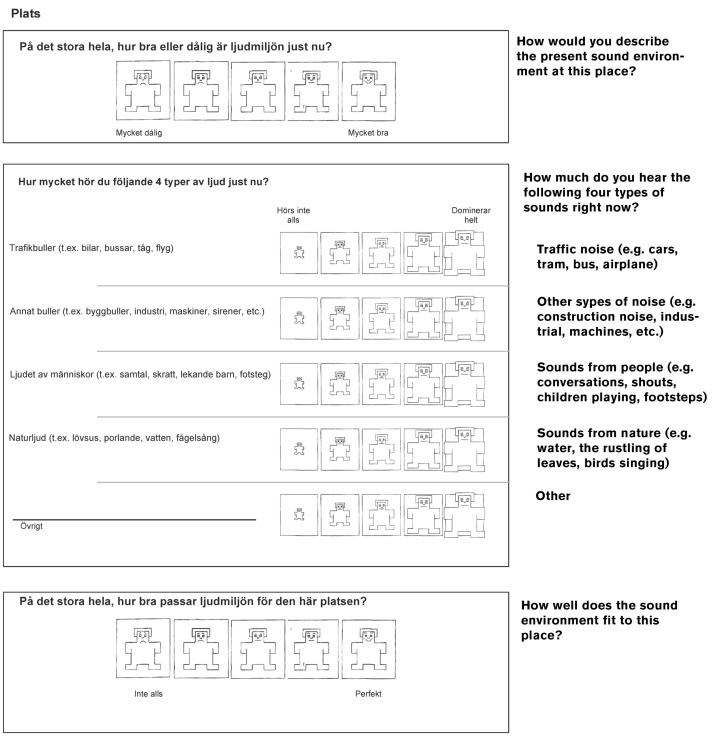
Soundwalk questionnaire.

Based on a series of consistent protocols and research works carried out by different working groups reported in the Introduction [36,37,38,39,40], the authors of the present paper developed a survey with three questions (see Figure 1). The questions were divided in three topics: quality of the sound environment; sources heard; and appropriateness of the sound environment, in the sense of fitness. The survey was made in Swedish; for the present purpose, an English translation is added.

- How would you describe the present sound environment at this place? (Q1)
- How much do you hear the following four types of sounds right now? (Q2)
- How well does the sound environment fit to this place? (Q3)

The survey incorporated the Bradley–Lang scale [70], explained in the Introduction. The scale was conceived of, among others, for children assessment, using the SAM.

The valence dimension was used for questions Q1 and Q3 regarding the quality and the appropriateness of the sound environment. The dominance dimension was used for question Q2 studying the different sound sources heard. When giving verbal instructions at the beginning of the soundwalk, words like “good”, “bad”, “perfect”, “happy”, “sad”, “heard”, “not heard”, “domination”, “being important”, and “not important” were included to describe the endpoints of the pictorial scales. Moreover, in the physical questionnaire, a short guidance of verbal assessment endpoints was included. Translated to English they were: “very bad” (1) and “very good” (5) for question Q1; “not hear at all” (1) and “dominates completely” (5) for question Q2; and “not at all” (1) and “perfectly” (5) for question Q3.

### 3.3. Soundwalk: Route and Sites’ Description

The district where the soundwalk was carried out was well known by the participants, making it possible to develop the soundwalk route in sound environments with which children felt familiar.

**Figure 2:**
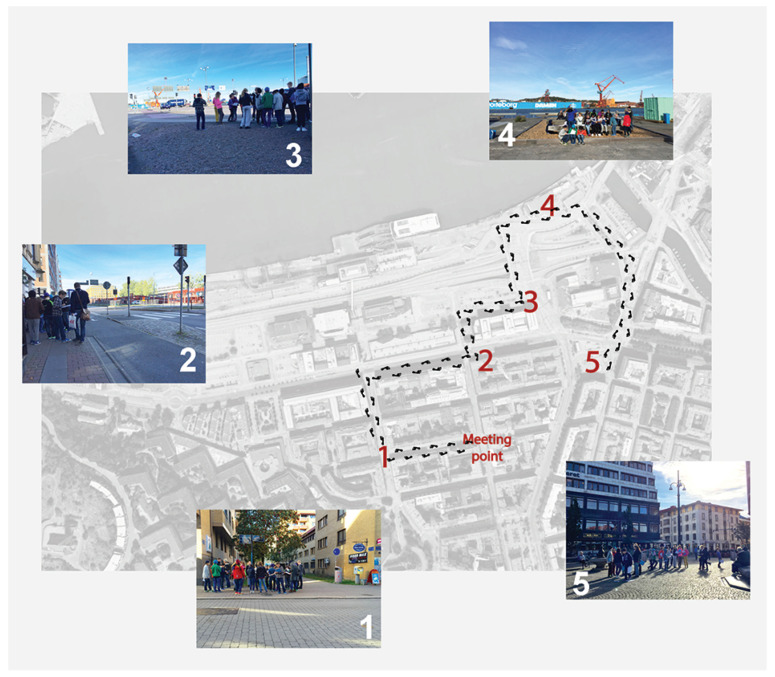
Soundwalk map: sites.

The route and sites were designed together with the district representative of the municipality, as well as with people working at the Environmental Office of the city of Gothenburg. The route (Figure 2) responded to the interest of the city in terms of assessment of the current situations and future urban developments. Different conditions were used to select the sites, explained in Table 1, together with a brief description and the latest available data on traffic from Gothenburg city [81]. In order to check the viability of the route and the sites, the route and the sites were first evaluated by the team in charge of performing the soundwalk.

### 3.4. Procedure

The children received the questionnaire and the instructions to carry out the soundwalk at the meeting point. They were instructed to listen for a moment before answering the questionnaire and to be an active listener, paying attention to details. The children were accompanied by their teacher, among other adults.

Both sound recordings and acoustic indicator data were captured using an in-house-developed acquisition software TAMARA (Lars Hansson in the Division of Applied Acoustics at Chalmers University of Technology, Gothenburg, Sweden) [83] with an appropriate microphone and a B&K 2260 sound level meter. The operator followed the soundwalk and recorded 3 min audio samples at each site. The sample rate used was 51,200 Hz, and results were presented in third octave bands. The post-processing steps were made in the software MATLAB ( The Mathworks, Inc., Natick, MA, USA) and SPSS v.23 (IBM, Armonk, NY, USA).

In order to test our prior hypothesis about the suitability and relevance of such a type of study of urban sound environment assessment by children, analyses were made mainly by comparison of sites, pairing questions as quality and appropriateness of the sound environment, and by studying the influence that the sound sources heard had on the sound environment assessment. A Kolmogorov–Smirnov test was run to determine the normality of the data, indicating that the data did not follow a normal distribution in either of the questions, always *p* < 0.001. Spearman correlation, showing the strength of dependence, was used to study the data. The Friedman test was used to detect differences between sites, as the alternative non-parametric test for one-way repeated measures Analysis Of Variance (ANOVA). In order to examine where the differences were occurring, a post-hoc Wilcoxon signed-rank test was used. Further analyses were intended, especially regarding the prediction of the sound environment assessment from the noise sources present; however, non-normality and the small sample size were limitations in this respect.

### 3.5. Acoustic Parameters

A list of acoustic parameters (noise indicators) and their values for each site, derived from the post-processing, is shown in Table 2. Apart from the equivalent A-weighted sound pressure level, the table includes three different percentiles of A-weighted sound pressure level, reflecting the sound pressure level exceeded for 10% (LA10), 50% (LA50), and 90% (LA90) of the measurement period. LA10 represents the peak noise level and LA90 the background noise level. Since busy traffic and trams were present at certain sites and the area was close to the river with maritime traffic (boats and ferries), equivalent C-weighted sound pressure levels are also presented.

Site 4 presented the lowest equivalent sound pressure level ($L_{Aeq}$), while the highest difference between $L_{Aeq}$ and $L_{Ceq}$ (15.1 dB), indicating a low frequency characteristic, was concluded to be due mainly to the closeness to the ferry stop.

## 4. Results

### 4.1. Initial Valuation of the Questionnaire Data

**Figure 3:**
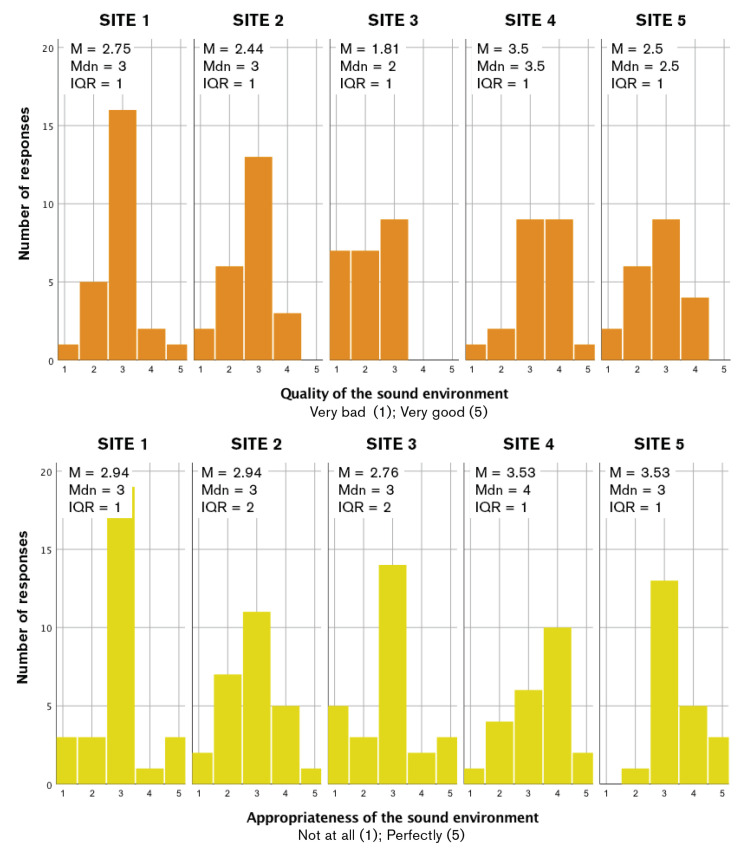
Histograms per site: quality (Q1) (**top**) and appropriateness (Q3) (**bottom**) of the sound environment. Mean (M), Median (Mdn), and Interquartile Range (IQR) values included.

The histograms for questions Q1 and Q3 (Figure 3) showed slightly skewed distributions in most cases (here indicated by differences between the Mean value, M, and Median value, Mdn). This gave a first indication on the relationship between the questions about quality (Q1) and appropriateness (Q3), i.e., the skewness tended to follow the same direction when comparing pairs from these two questions, except for Site 5. This is investigated further below. Furthermore, the resulting Interquartile Range (IQR) values reflected a greater agreement among the children on the sound environment quality (Q1) than on the appropriateness (Q3). Furthermore, the resulting mean values showed that the children in general rated the sound environment appropriateness to the site (Q3) higher than its sound quality (Q1).

Site 4 was judged as the best place regarding its sound environment. It had the lowest equivalent sound pressure level ($L_{Aeq}$); however, it presented a significant low-frequency character, reflecting the complexity of sound environment evaluation.

### 4.2. Relationship between Acoustic Parameters and Sound Environment Assessment

In the present study, Spearman correlations were performed to evaluate if there was a relationship between acoustic indicators and the evaluation of the sound environment. The results showed that there was a moderate negative correlation in all cases (Table 3), which meant that when the noise levels were lower, the children considered that the sound environment was better. Significant correlations were found between the quality of the sound environment (Q1) and $L_{A90}$ (*r_s_* = −0.38, *n* = 150, *p* < 0.001) and its appropriateness (Q3) and *L*${}_{A90}$ (*r_s_* = −0.24, *n* = 150, *p* < 0.001). Peak noise level ($L_{A10}$) and equivalent sound pressure level ($L_{Aeq}$) showed a negative relationship with Q1 and Q3 as well, but at a lower significance (*p* < 0.05). C-weighted sound pressure levels ($L_{Ceq}$) reflected as well a negative correlation with Q1 (*p* < 0.05).

### 4.3. Quality and Appropriateness of the Sound Environment Assessment and Sites

In the present study, the Spearman correlation was used to determine the relationship (dependency force) between the sound environment quality (Q1) (very bad/very good) and the appropriateness (Q3) (not at all/perfectly), showing a moderate positive correlation (*r_s_* = 0.26, *p*≤ 0.007) in the children’s responses. To examine whether children were able to differentiate between the soundscapes of different sites within a soundwalk methodology, a Friedman test was performed. The dependent variable was “quality of the sound environment”, and the independent variable was “site”, which consisted of five sites. There was a statistically significant difference in the description of the sound environment according to the site, $X^{2}\left(4\right)=25.180$, *p* < 0.001. A post hoc analysis was performed with Wilcoxon signed rank tests (Bonferroni correction), which resulted in a significant level (*p* < 0.005). There were significant differences between certain qualities of the sound environment pairwise: Sites 1 and 3, sites 2 and 3, Sites 2 and 4, and Sites 3 and 4.

### 4.4. Sound Source Dominance

Based on the results, the use of Spearman correlation demonstrated that traffic dominance as a sound source had a strong positive relationship with acoustic indicators as $L_{A90}$ (r*_s_* = 0.69, n = 150, *p* < 0.001), $L_{A10}$ (r*_s_* = 0.43, n = 150, *p* < 0.001) and $L_{Aeq}$ (r*_s_* = 0.39, n = 150, *p* < 0.001).

Regarding the sound environment assessment, a strong negative relationship between the quality of the sound environment and the dominance of traffic as a sound source was shown (r*_s_* = −0.54, *p* < 0.001), meaning that traffic source dominance might be a good predictor for acoustic quality. Moreover, traffic source dominance was correlated with traffic volume (vehicles/day) in a positive relation (r*_s_* = 0.287, n = 150, *p* < 0.001).

The latter may have more to do with the children’s impression of the spatial configuration of the area and the presence of much traffic than with the actual magnitude of the sound level: a moderate negative correlation between the volume of traffic and the quality of the sound was shown (r*_s_* = −0.221, *p* = 0.017), which meant that more traffic was linked with a lower quality of the sound environment. However, very different traffic volumes presented the same $L_{Aeq}$ (63.9 dB); Site 3 had 22,320 vehicles/day and Site 4 had 7380 vehicles/day on the road closest to the site).

Regarding other sound sources, a weak negative relationship was found between the dominance of other sound sources, e.g., mechanical sounds, and the quality of the sound environment (r*_s_* = −0.21, *p* = 0.028).

On the relationship between the sound environment appropriateness (Q3) and the dominance of traffic, a negative correlation was present (r*_s_* = −0.241, *p* = 0.007), however weaker than the corresponding result for the quality (Q1). A positive correlation was found with the dominance of nature sound sources (r*_s_* = 0.25, *p* = 0.005).

**Figure 4:**
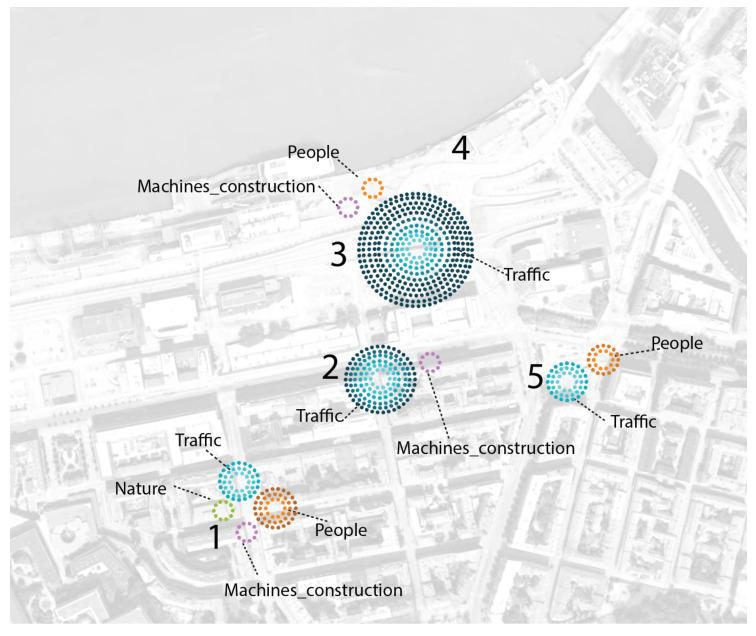
Site map: frequencies of sources heard. Each circle corresponds to 10% of the participants giving values of four (dominates) and five (dominates completely) on a scale 1–5 (Site 4 had no dominating sound source type).

Figure 4 presents a graphical tool for visualizing the domain responses of the sound sources. Each circle corresponds to 10% of the participants giving clear dominance values (pictograms corresponding to the fourth and fifth dominance on the 1–5 scale) to that particular noise source. We can see, for example, that Site 4 had a sound environment where none of the sound sources was marked as dominant. Site 3 was clearly dominated by traffic noise, which 100% of the children reported as dominant. Site 1 seemed to be more balanced, reflecting the presence of the four sound sources, although with a greater presence of traffic noise and people.

## 5. Discussion

Our findings suggested that the following analyzed variables could be used to indicate the quality of the sound environment as judged by children:

- Traffic dominance ratings appeared as the best predictor: when traffic dominated as a sound source, the evaluated quality of the sound environment was judged as worse.
- Traffic volume appeared as a plausible predictor: the higher the traffic volume, the lower the sound environment quality.
- About noise levels, the best environmental sound quality predictor appeared to be the background noise level ($L_{A90}$): children tended to rank the sound environment as slightly better when lower levels of background noise were present.

The results presented certain similarities, as well as discrepancies, in relation to those from other sound environment studies in urban areas, where different groups of adults were targeted (regular visitors, acousticians, architects, etc.). For example, technological sounds, including road traffic, have been correlated with poor soundscape quality [53]. Moreover, adjectives such as “chaotic” and “annoying”, implying poorer sound quality, have been related to traffic dominance [36]. $L_{A50}$ has been highlighted as the best statistical indicator for the quality of the sound environment prediction among adults [36,53,84], showing high correlation levels. In the present study, the correlation existed, however not as strongly as in the reported studies on adults. In our study, the background noise level appeared as a more important indicator for environmental sound quality. The reason for this difference may be due to an intrinsic difference between children and adults concerning the perception of the sound environment; however, more data would be needed to support that hypothesis.

Concerning the concept ratings on the “quality” and “appropriateness” of the sound environment, the results from the study showed that there could be clear differences, with a weak dependence of 26% (positive association). For example, the appropriateness of the sound environment was rated higher than its quality for Site 5 (a square surrounded by heavy traffic and a large presence of trams) and for Site 3 (a vacant lot facing a motorway). Site 3 had the lowest ratings on both questions among the five sites studied. Among adults, strong correlations have been found, implying that a high level of the appropriateness of the sound environment attribute is needed to perceive a high sound environment quality [36]. However, the concept of the appropriateness of the sound environment has been highlighted in adult studies as a problematic concept [37]. In addition, appropriateness has been related to the potential or current activities carried out in the space, rather than the relationship with the site itself [61]. The conflict of understanding the actual meaning of appropriateness of the sound environment is expected to be similar or even more pronounced within children. In this case, it could be that children see the sites as similar ones: traffic is present; it is an urban area, etc. Methodologically, it could be the limited understanding among children of the meaning of sound environment appropriateness and/or the absence of specific activities related to it. Furthermore, children usually have no decision-making power or are not consulted about their environment and may not know how to evaluate it.

Children have been highlighted as a “vulnerable group” for environmental exposure [7] including noise exposure [4]. The detriment of their physical and psychological skills has been proven in numerous studies [26,27,28,29,30,31,32]. The fact that the outdoor sound environment also affects children’s ability for interaction and development should be considered. Hence, the incorporation of children into sound environment investigations could generate new knowledge about children’s understanding of the sound environment, and sound environment policies can be developed from and for children, assisting in the urgency of putting children at the center of efforts for sustainable development [15]. The sound environment of the City of Gothenburg is constantly under study; however, we encourage the city municipality to incorporate children both in the sound environment assessment and in the consequence analyses of the different studies, e.g., concerning the current legislation on city parks.

Moreover, we must not forget that children are an active part of the society and should be treated in this way. Learning to listen in a way that they can take action is pursued, and soundwalks seem to have the potential to do so.

## 6. Conclusions

According to the literature, the inclusion of children in the study of the sound environment has been pointed out as relevant. They are considered a “vulnerable group” who are clearly at a disadvantage considering their reduced control over how and where they play, go to school, and live [7]. If we promote effective noise policies under the umbrella of urban planning policies, the public needs to better understand sound exposure and its consequences [20]. With this type of evaluation, we want to highlight the importance of including children and other vulnerable groups in society in sound environment policies. Swedish environmental noise legislation considers the general public, and no special reference is made to children, except on limit values for noise in schoolyards [85].

The present study aimed to test the concept of soundwalks in children and to analyze the sound environment of different sites in Gothenburg, from children’s view-point. Both individual assessment of the sound environment and acoustic data were collected. Statistically significant results were shown for the negative correlation between measured background noise level ($L_{A90}$) and perceived sound environment quality. Hence, the study showed that the concept of children’s soundwalks can be included in the study of the sound environment. Furthermore, it was shown that having knowledge of the dominance of sound sources could help to understand how children would evaluate their sound environment.

However, limitations were present, as described in the paper. The most evident were the small number of participants and the similarity of the places evaluated with respect to sound pressure levels and the presence of traffic.

Future lines of research will be associated with the limitations found, including further testing and questionnaire validation, with a larger number of sites and variability, along with a larger number of children with different backgrounds. In this soundwalk, three minute acoustic measurements were performed at each site at the same time as the children evaluated the sound environment. The children were familiar with the sites, and the sound environments did not show large changes in character during the time at each site. Moreover, there was a need to perform short measurements at each site to avoid the sounds from the children being recorded and to prevent the children from getting tired of waiting and losing interest. However, further studies of the influence of measurement duration within soundwalks are recommended. In addition, further exploration of a four point pictorial scale for the sound environment evaluation might be of interest as it could exclude the central/neutral trend.

## Figures and Tables

**Table 1:** Sites’ description and selection conditions.

Site	Description	Traffic Amount	Site Selection Criteria ^1^
1. Värmlandsgatan and Tredje Långgatan corner	Street crossing with one of the intersections being pedestrian	5400 veh/day in 2013 (5% heavy)	High noise levels
2. Första Långgatan and Nordhemsgatan corner	Sidewalk facing several shops and restaurants located on a wide highway dedicated to private and public transport, consisting of 2 lanes, two centrally located tram lines, and two other lanes; a two-story parking building is located across the road	5840 veh/day in 2013 (6% heavy)	High noise levels
3. Järnsvågsgatan between Masthamnsgatan and E45 exit	Vacant lot located on a corner facing a crossroads, including a tunnel exit, from one of the main city tunnels	52,380 veh/day in the tunnel in 2016; Järnsvågsgatan had 22,320 veh/day in 2016 (no data on the percentage of heavy vehicles)	High noise levels + urban transformation
4. River front area at Emigrantsvägen and Masthamnsbron	Open space with no specific activities between the city river and a road	7380 veh/day in 2016 (no data on the percentage of heavy vehicles)	High noise levels + urban transformation
5. Järntorget	Very popular square, with numerous shops and restaurants. It is one of the main city hubs of public transport and a meeting place. Private vehicles, buses and trams are present all the time. A fountain is located in the center of the square	16380 veh/day in 2016 (no data on the percentage of heavy vehicles)	High noise levels + popular place

^1^ Urban transformation: sites are part of the new development strategy of Gothenburg city [80]. High noise levels: environmental outdoor noise levels for $L_{den}$ (day-evening-night noise level) above 55 dB and above 50 dB for $L_{night}$ (night noise level) [82]. Popular place: well-known spaces used by citizens and tourists.

**Table 2:** Acoustic parameters for the five sites.

Site	1	2	3	4	5
$L_{Aeq}$ (dB)	64.7	63.9	63.9	58.3	60.0
$L_{A10}$ (dB)	67.9	65.9	66.1	60.7	62.8
$L_{A50}$ (dB)	62.5	61.2	62.2	57.3	57.7
$L_{A90}$ (dB)	56.5	58.7	59.7	54.1	55.6
$L_{Ceq}$ (dB)	72.5	73.6	77.8	73.4	71.0

**Table 3:** Spearman correlation between acoustic indicators and the sound environment assessment (** correlation is significant at the 0.01 level; * correlation is significant at the 0.05 level). The mean values of acoustic indicators are incorporated.

Acoustic Indicators	Q1. Quality of the Sound Environment	Q3. Appropriateness of the Sound Environment	Mean Value
$L_{Aeq}$ (dB)	−0.185 *	−0.217 **	62.2
$L_{A10}$ (dB)	−0.228 *	−0.212 *	64.7
$L_{A50}$ (dB)	−0.228 *	−0.212 *	60.2
$L_{A90}$ (dB)	−0.378 **	−0.237 **	56.9
$L_{Ceq}$ (dB)	−0.214 *	−0.171	73.7
